# The prevalence and associated factors of prenatal depression and anxiety in twin pregnancy: a cross-sectional study in Chongqing, China

**DOI:** 10.1186/s12884-022-05203-y

**Published:** 2022-11-26

**Authors:** Ying Zhou, Jingui Huang, Philip N. Baker, Bizhen Liao, Xinyang Yu

**Affiliations:** 1 Department of Obstetrics, the First Affiliated Hospital of Chongqing Medical University, No. 1, Youyilu Street, Yuzhong District, 400016 Chongqing, China; 2 Department of Oncology, Chongqing University Cancer Hospital, 400030 Chongqing, China; 3 College of Life Sciences, University of Leicester, LE1 7RH Leicester, UK

**Keywords:** Twin pregnancy, Depression, Anxiety, Maternal mental health, Prevalence

## Abstract

**Background:**

Pregnant women expecting twins are more likely to experience stress, which can lead to anxiety and depression. Our aim was to investigate the prevalence of prenatal anxiety and depressive symptoms in women with twin pregnancies and the associated factors.

**Methods:**

In a cross-sectional survey, 210 women with twin pregnancies who satisfied the inclusion and exclusion criteria in two tertiary centers in Southwestern China were asked to complete a basic information form, the Self-Rating Anxiety Scale (SAS) and the Self-Rating Depression Scale (SDS). To compare statistics with normal distribution in distinct characteristic groups, a paired t-test, and one-way ANOVA were utilized. Binary logistic step regression was used to analyze the associated factors of antenatal anxiety and depressive symptoms.

**Results:**

The 210 women with twin pregnancies (age = 30.8 ± 4.2 years) were between 7 and 37 gestational weeks (29.2 ± 1.2 weeks), were typically well-educated (72.4% had a post-high-school degree), and reasonably affluent (88.1% were above the low-income cutoff). Among them, 34.8% had symptoms associated with clinical levels of anxiety, and 37.1% had symptoms indicating possible depression. The prevalence of co-morbid anxiety and depressive symptoms was 24.3%. Binary stepwise logistic regression analysis showed that previous health status and sleep disturbance during pregnancy were the associated factors of anxiety symptoms in women with twin pregnancies (*P* < 0.05), whereas age, previous health status, negative life events, and physical activity during pregnancy were the associated factors of depressive symptoms in women with twin pregnancies (*P* < 0.05).

**Conclusion:**

About one-third of women with twin pregnancies had symptoms of anxiety or depression; these were most strongly predicted by some modifiable factors, suggesting that early preventive mind-body interventions may be a promising strategy to protect against mental health issues for women with twin pregnancies.

## Background

With the increasing maternal age at conception and the development of assisted reproduction techniques (ART), the incidence of twin births has dramatically risen over recent decades [1, 2]. A population-based report found that the twin birth rate in the United States increased from 1.89% to 1980 to 3.19% in 2020 [3, 4]. China’s twin birth rate increased from 2.84% to 2012 to 3.22% in 2020 [5]. A recent report compared the data collected in 112 countries from 1980 to 1985 and 165 countries from 2010 to 2015 and found that the global twin birth rate has increased from 9.1 to 1000 to 12 per 1000, which means one of every 42 children born on earth is a twin [6]. Because of the increased twin birth rate all over the world, more attention should be devoted to the health of the population of women with twin pregnancies.

Significant physiological changes, particularly cardiovascular and glucose metabolic changes, may pose considerable challenges to women with twin pregnancies [7, 8]. Correspondingly, pregnant women with twins are more likely to develop hypertensive disorders of pregnancy, gestational diabetes, and other maternal complications [9, 10]. The increased incidence of offspring deaths and morbidity in twin pregnancies, as compared to singleton pregnancy [11, 12], was attributed to preterm delivery [13] and intrauterine growth restriction [14]. For this reason, a twin pregnancy is classified as a high-risk pregnancy. Furthermore, the increased frequency of examinations, prolonged hospitalization [15], and the increased expenditure budget for bringing up two children place an additional financial burden on women pregnant with twins and their families [16]. All of these unavoidable factors may impact the mental health of women who have conceived twins. Hence, evaluating the psychological condition of women with twin pregnancies is of considerable importance.

Only a few studies have been undertaken on the prevalence and associated factors of anxiety and depressive symptoms in women with twin pregnancies. Wang et al. [17] reported that prenatal stress in twin pregnancies increases as the pregnancy progresses. Prenatal stress may raise the chance of adverse emotional outcomes such as anxiety and depressive symptoms in women with twin pregnancies [18]. In 1995, Thorpe et al. compared 147 women pregnant with twins and 11,061 women pregnant with singletons. They found that the prevalence of depressive symptoms is the same, 31% and 26%, at 18 and 32 weeks of gestation [19]. In 2013, major depression was reported in 33.3% (*n* = 51) of women with twin pregnancies, and this depression was associated with higher stress levels and a lack of social support [20]. A more comprehensive study utilizing larger sample sizes is needed.

The mental health of pregnant women may be related to pregnancy outcomes. High- stress levels in women with twin pregnancies may increase the risk of premature rupture of membranes and preterm birth [17, 21]. The severity of prenatal depressive symptoms has been associated with low Apgar scores of boys in twin neonates [22]. A systematic review has shown that maternal prenatal anxiety and depression have a long-lasting negative influence on the neurodevelopment of children and adolescents [23]. Furthermore, women with higher levels of antenatal anxiety are at increased risk of elevated levels of postnatal anxiety [24]; moreover, postnatal depression may be the continuation of prenatal depression [24, 25]. In conclusion, twin pregnancies may be associated with poor psychological consequences [26]. The financial burden of untreated perinatal mental disorders is still considerable [27]. Thus, negative prenatal mood and disorders are perceived as significant patient safety issues, public health problems, and preventable causes of maternal and infant mortality, which is of concern to healthcare providers and policymakers [28].

Previous studies on pregnant women’s psychological status mainly focused on women pregnant with singletons. However, the mental health of women pregnant with twins has rarely been investigated. As far as we know, the prevalence of anxiety symptoms and co-morbid anxiety and depressive symptoms in twin pregnancies has not been reported. So, the study’s objective was to conduct a cross-sectional study to investigate the prevalence of prenatal anxiety and depression symptoms in women with twin pregnancies and the potential associated factors, including negative life events, sleep disturbance, and physical activity. This study’s findings will be valuable for identifying the high-risk population of antenatal depression and anxiety for women pregnant with twins and developing preventative interventions.

## Methods

### Design

The study adopted a cross-sectional design and a convenient sampling method. Data were collected at the First Affiliated Hospital of Chongqing Medical University and the Chongqing Health Center for Women and Children from early March to late September 2021. Participants were recruited during routine prenatal examinations at outpatient services designed for women pregnant with twins. After reviewing the medical records, women with twin pregnancies who met the inclusion criteria were invited to participate in the study until the required sample size was reached. Two trained investigators explained the study’s objective, content, and cooperation to the participants. Then participants signed the informed consent and completed several electronic questionnaires. The ethics committee of the First Affiliated Hospital of Chongqing Medical University approved the study (2020 − 199).

### Sample size calculation

The sample size was calculated by assuming that the standard deviation of SDS in women with twin pregnancies would be 4.3 in China [29]. If the error is less than 0.6 and α = 0.05, the calculated sample size is *n*= (Z_1−α/2_*σ/δ) ^2^= (1.96*4.3/0.6) ^2^=198.This result would require the sample size to be roughly 220 to achieve a 10% loss rate for the questionnaire.

### Participants

Participants were required to be at least 18 years old, be diagnosed with twin pregnancy by B-scan ultrasonography before 12 ~ 14 weeks of pregnancy, be able to read Chinese, and be willing to participate in the study. Diagnosed with severe organic or congenital diseases, fetal reduction, and pre-pregnancy psychiatric illnesses such as bipolar and schizophrenia were excluded. The study included participants receiving psychotherapy or medication because of prenatal anxiety or depression. Two cases of fetal reduction and one request for termination of pregnancy were excluded, and seven potential participants declined due to lack of time.

## Materials

### Basic information form

Participants provided detailed demographic information, such as their age, pre-pregnancy BMI (kg/m^2^), education, occupation, and monthly per capita household income (RMB, renminbi, Chinese yuan). The medical conditions, including the history of psychiatric disorders and previous pregnancy disorders, were collected through medical records. The self-compiled questions were used to evaluate previous health status, depression history, negative life events, sleep disturbance (i.e., difficulty in falling asleep, waking up easily at night or waking up early, insomnia, daytime fatigue, or sleepiness) [30] and regular physical activity (i.e., moderate-intensity aerobic or strength exercise average 20–30 min per day) [31] during pregnancy.

### Self- rating anxiety scale

The 20-item Self-Rating Anxiety Scale (SAS) [32] was compiled by American doctor William W.K. Zung in 1971. It is widely used and validated in assessing anxiety symptoms [33]. SAS is a four-point Likert scale ranging from 1 (no/a little of the time) to 4 (most of the time/all the time). Higher scores indicate more severe anxiety symptoms. The standard score equals the sum of scores from 20 items multiplied by 1.25. In this study, the standard score below 50 indicates a normal range of anxiety symptoms; 50–59 mild; 60–69 moderate; and over 69 indicates extreme anxiety symptoms [34]. The Cronbach’s alpha coefficient of the scale was reported as 0.84 [35]. In the current study, Cronbach’s alpha was 0.73.

### Self-Rating Depression Scale

The Self-Rating Depression Scale (SDS) [36] by Zung was used to assess depressive symptoms over the past month. It has been demonstrated as a valuable and effective tool for detecting depressive symptoms in pregnancy [37]. The scale had 20 items, each of which was scored on a four-point scale ranging from 1 (no/a little of the time) to 4 (most of the time/all the time). The standard score equals the cumulative score of 20 items multiplied by 1.25. In this study, the standard score below 53 indicates a normal range of depressive symptoms; 53–62 mild; 63–72 moderate; and over 72 indicates extreme depressive symptoms [34]. The Cronbach’s alpha coefficient of the scale was reported as 0.86 [35]. The current sample has an internal consistency of 0.85.

### Statistical analysis

We analyzed the responses of 210 cases of women with twin pregnancies after excluding ten respondents due to incorrect or incomplete responses. All statistical analysis was performed with SPSS 26.0 software. First, descriptive statistics were used to review the general data; measurement data was expressed in mean ± SD, and count data were expressed in rates. A paired t-test and one-way ANOVA were used to compare the data with normal distribution in different characteristic groups. Mann-Whitney U and Kruskal-Wallis H tests were used to compare the data with skewed distribution. The above *P* < 0.2 factors in the corresponding analysis were included in the binary logistic stepwise regression. Multivariate logistic regression analysis assessed the associations of prenatal anxiety and depressive symptoms with different factors. *P* < 0.05 was considered to indicate statistical significance. Data analysis was based on SAS and SDS scoring criteria.

## Results

### Baseline characteristics of women with twin pregnancies

The participants were women pregnant with twins aged between 21 and 46 years (30.8 ± 4.2) with a mean pre-gravid Body Mass Index (BMI) of 23.0 kg/m^2^ (± 3.9, range 14.8–38.1). It was determined that 72.4% of the women had an educational level above high school, and 83.3% belonged to the middle- and high-income groups (monthly per capita household income over 4,000 yuan). The average gestational age of women with twin pregnancies was 29.2 (± 1.2, range 7–37), and most were assisted reproductive (65.2%) and dichorionic diamniotic (80.5%) twin pregnancies. The baseline characteristics of the population are shown in Table 1.

**Table 1:** The level of anxiety and depressive symptoms in different characteristic groups

Variable		N (%)	SAS	t/F Value	P	SDS	t/F Value	P
**Maternal age (year)**				3.04	0.03		1.96	0.12
	< 25	14(6.67)	50.63 ± 5.34			54.20 ± 10.89		
	25~29	64(30.48)	46.99 ± 5.51			51.04 ± 9.23		
	30~34	93(44.28)	47.30 ± 6.98			50.08 ± 9.33		
	≥ 35	39(18.57)	44.81 ± 6.89			47.76 ± 8.32		
**Pre-gravid Body Mass Index (kg/m**^**2**^**)**				0.44	0.65		0.46	0.63
	< 18.5	15(7.14)	45.50 ± 6.92			48.50 ± 12.25		
	18.5~24	123(58.57)	47.17 ± 6.26			50.66 ± 8.98		
	≥ 24.0	72(34.29)	46.91 ± 6.70			49.81 ± 9.22		
**Education**				2.02	0.05		1.92	0.06
	≤Senior high school	58(27.62)	48.43 ± 6.76			52.20 ± 9.34		
	>Senior high school	152(72.38)	46.41 ± 6.40			49.46 ± 9.20		
**Monthly per capita household income (yuan)**				2.07	0.13		2.44	0.09
	< 4000	25(11.90)	48.95 ± 5.84			53.50 ± 7.28		
	4000~8000	97(46.20)	47.26 ± 6.63			50.50 ± 9.53		
	> 8000	88(41.90)	46.08 ± 6.55			48.96 ± 9.30		
**Previous health status**				-6.21	0.00		-4.69	0.00
	Good	158(75.4)	45.48 ± 5.75			48.57 ± 8.80		
	Worse	52(24.76)	51.47 ± 6.80			55.22 ± 9.05		
**Depression history**				4.33	0.00		2.64	0.01
	Yes	8(3.81)	56.41 ± 8.88			58.59 ± 11.72		
	No	202(96.19)	46.59 ± 6.17			49.88 ± 9.06		
**Gestation age (week)**				1.60	0.11		1.06	0.29
	< 28	73(34.76)	45.98 ± 6.91			49.28 ± 9.94		
	≥ 28	137(65.24)	47.49 ± 6.30			50.71 ± 8.93		
**Parity**				0.96	0.34		1.30	0.20
	Primiparous	164(78.10)	47.20 ± 6.50			50.66 ± 9.48		
	Multiparous	46(21.90)	46.14 ± 6.72			48.64 ± 8.51		
**Mode of conception**				-1.24	0.22		-1.71	0.09
	Spontaneous conceived	73(34.76)	46.20 ± 5.84			48.72 ± 8.90		
	Assisted reproduction	137(65.24)	47.37 ± 6.88			51.01 ± 9.44		
**Planned pregnancy**				-1.31	0.19		-1.31	0.19
	Yes	188(89.52)	46.76 ± 6.53			49.93 ± 9.13		
	No	22(10.48)	48.69 ± 6.53			52.67 ± 10.52		
**Type of pregnancy**				0.52	0.60		0.65	0.52
	Dichorionic diamniotic	169(80.48)	46.75 ± 6.68			50.05 ± 9.44		
	Monochorionic diamniotic	31(14.76)	47.62 ± 6.49			50.04 ± 9.26		
	Monochorionic monoamniotic	10(4.76)	48.50 ± 3.94			53.50 ± 6.61		
**Negative life events**				2.81	0.01		4.10	0.00
	Yes	29(13.81)	50.09 ± 5.94			56.55 ± 9.17		
	No	181(86.19)	46.46 ± 6.51			49.20 ± 8.93		
**Sleep disturbance**				4.55	0.00		3.27	0.00
	At least one	59(28.10)	50.10 ± 6.99			53.50 ± 9.35		
	None	151(71.90)	45.74 ± 5.95			48.93 ± 8.98		
**Physical activity regularly**				-2.62	0.01		-3.58	0.00
	Yes	89(42.38)	45.60 ± 6.15			47.61 ± 8.90		
	No	121(57.62)	47.96 ± 6.67			52.13 ± 9.15		

*N* number, *SDS * Self-Rating Depression scale, *SAS * Self-Rating Anxiety scale, *P * p-value

## Prenatal anxiety and depressive symptoms

**Fig. 1:**
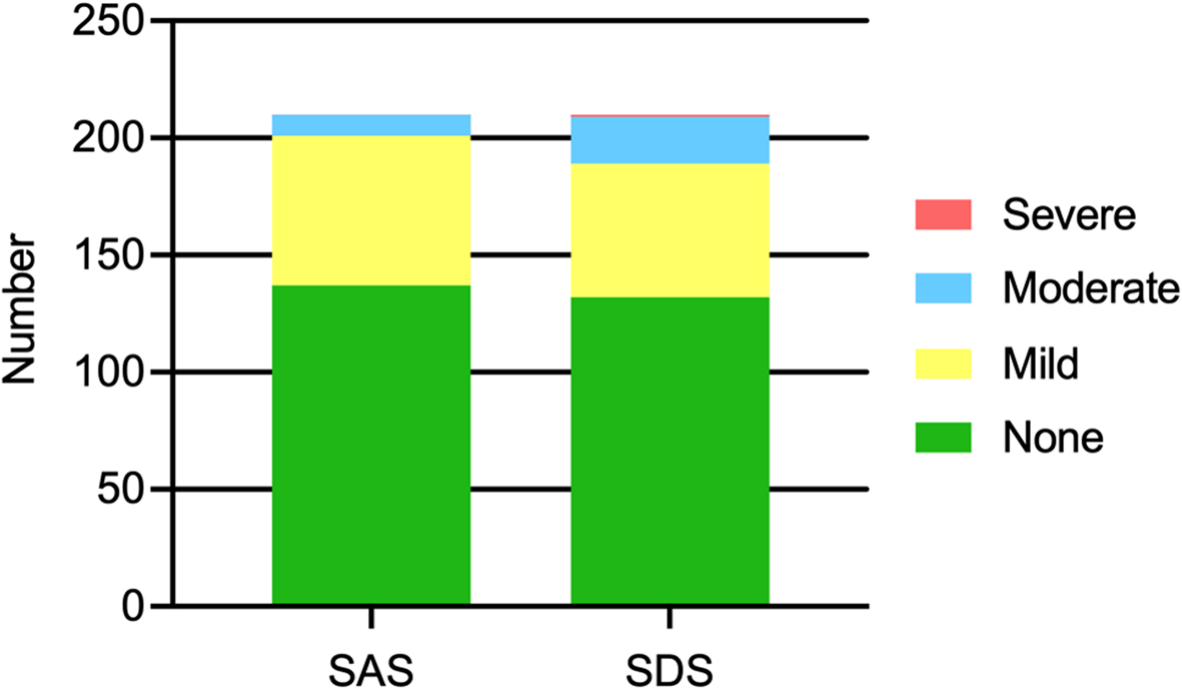
The number of women with twin pregnancies in different degrees of anxiety and depressive symptoms. SDS: Self-Rating Depression scale; SAS: Self-Rating Anxiety scale

The SAS standard score was 47.0 ± 6.5 in women with twin pregnancies. In the sample, 34.8% (95%CI = 28.4–41.7, *n* = 73) had symptoms associated with clinical anxiety levels. 137 women were non-anxious (65.2%), 64 women were mildly anxious (30.5%), nine women were moderately anxious (4.3%), and none were severely anxious. On the SDS scale, women with twin pregnancies had an average score of 50.2 (± 9.3), and 37.1% (95%CI = 30.7–44.1, *n* = 78) reported depressive symptoms. Among them, 132 were non-depressed (62.9%), 57 were mildly depressed (27.1%), 20 were moderately depressed (9.5%), and one was severely depressed (0.5%). The prevalence of co-morbid anxiety and depressive symptoms was 24.3% (95%CI = 18.8–30.8, *n* = 51). (See Fig. 1)

## Associated factors of prenatal anxiety and depressive symptoms

Good previous health status was associated with a decreased risk of prenatal anxiety symptoms in pregnant women with twins (OR 0.2, 95%CI 0.1–0.4). Sleep disturbance was associated with an increased risk of prenatal anxiety symptoms in women with twin pregnancies (OR 3.1, 95%CI 1.6–6.0). (See Table 2)

**Table 2:** Binary stepwise logistic regression analysis of anxiety symptoms in women with twin pregnancies

Variable		Anxiety Symptoms Number (%)	B	Wald	df	P	OR	95%CI	
								Lower	Upper
Previous health status	Good	40(54.79)	-1.61	20.86	1	0.00	0.20	0.10	0.40
	Worse	33(45.21)					1.0		
Sleep disturbance	At least one	32(43.84)	1.12	10.94	1	0.00	3.07	1.58	5.97
	None	41(56.16)					1.0		
Constant			0.19	0.36	1	0.55	1.21		

*B * Regression coefficients, *df * degree of freedom, *P * p-value, *OR * Odds Ratio, *CI * Confidence Interval

Maternal age less than 25 years (OR 3.9, 95%CI 1.2–13.1) and having negative life events in pregnancy (OR 3.3, 95%CI 1.4–7.7) was associated with an increased risk of prenatal depressive symptoms in women with twin pregnancies. Good previous health status negatively correlated with prenatal depressive symptoms (OR 0.4, 95%CI 0.2–0.8). Women with twin pregnancies who undertook physical activity regularly during pregnancy had fewer depressive symptoms (OR 0.4, 95%CI 0.2–0.8). (See Table 3)

**Table 3:** Binary stepwise logistic regression analysis of depressive symptoms in women with twin pregnancies

Variable		Depressive Symptoms Number (%)	B	Wald	df	P	OR	95%CI	
								Lower	Upper
Maternal age	< 25 year	9(11.54)	1.36	4.76	1	0.03	3.88	1.15	13.10
	≥ 25 year	69(88.46)					1.0		
Previous health status	Good	49(62.82)	-0.89	6.57	1	0.01	0.41	0.21	0.81
	Worse	29(37.18)					1.0		
Negative life events	Yes	17(21.79)	1.19	7.38	1	0.01	3.28	1.39	7.74
	No	61(78.21)					1.0		
Regular physical activity	Yes	23(29.49)	-0.89	7.36	1	0.01	0.41	0.22	0.78
	No	55(70.51)					1.0		
Constant			0.20	0.44	1	0.51	1.23		

*B * Regression coefficients, *df * degree of freedom, *P * p-value, *OR * Odds Ratio, *CI * Confidence Interval

## Discussion

There is growing research in the field of prenatal mental health. However, women with twin pregnancies as the vulnerable group to the mental disorder are still overlooked. To the best of our knowledge, this is the first study to investigate the overall prevalence of both depressive and anxiety symptoms in twin pregnancies with a comparatively large sample size. The key findings of this study were that 34.8% of women pregnant with twins were suffering from anxiety symptoms, and 37.1% of women pregnant with twins had depressive symptoms. The incidence of self-reported co-morbid anxiety and depressive symptoms in women pregnant with twins was 24.3%, higher than the previously reported prevalence in singletons (9.5%) [38]. In summary, according to this study, one in three women pregnant with twins was anxious or depressed.

In this study, the incidence of anxiety symptoms in women with twin pregnancies (34.8%) was significantly higher than that previously reported in women with singleton pregnancies (18.2-24.6%) [39]. Because the rate of maternal and fetal complications and neonatal adverse outcomes is higher in twin pregnancies than in singleton pregnancies [40], this may bring more stress regarding concerns about pregnancy and childbirth safety in women pregnant with twins [17]. The stress related to the adverse consequence may eventually cause anxiety symptoms [41]. The SAS score of women with twin pregnancies (47.0 ± 6.5) in our study was lower than in a 2014 study of the same region (52.9 ± 10.1, *n* = 60 )[29]. One plausible explanation for this reduced level of anxiety is that over the ensuing years since 2014, treatments for twin pregnancy complications have been developed (such as fetus endoscopy and fetoscopic laser ablation) that have improved neonatal survival rates [42], possibly reducing the anxiety experienced by women with twin pregnancies.

The high prevalence rate of depressive symptoms (37.1%) in this study is comparable to other studies. A recent multi-site study in mainland China found that the prevalence of prenatal depression among singleton pregnant women was 28.4% [43]. According to a systematic review and meta-analysis, the weighted prevalence of prenatal depression was reported to be 19.7% in Chinese singleton women [44]. The prevalence of depressive symptoms in women pregnant with twins is thus higher than in women pregnant with singletons in China. The SDS score of women with twin pregnancies (50.2 ± 9.3) was similar to the previous studies ( 50.6 ± 4.3) [29]. The overall level of depressive symptoms among twin pregnancy women remains high. This situation may be attributed to a lack of further evaluation and continuous monitoring [45] and poor access to quality mental health services [46, 47].

The psychological condition of women with twin pregnancies needs attention and intervention urgently. Exploring the factors associated with anxiety and depressive symptoms in pregnancies with twins may contribute to identifying the twin pregnancy population at high risk of developing anxiety and depression and benefit the selection of preventative interventions in prenatal care.

We found that sleep disturbance during pregnancy was associated with anxiety symptoms in women with twin pregnancies. Sleep disturbance may be caused by changes in lifestyle, physiology, or psychology during twin pregnancy[30]. Long-term sleep disturbance can lead to circadian rhythm disorder, affect the autonomic nervous system and limbic system (which is involved in mediating emotion), worsen mood vulnerability and instigate or exacerbate anxiety symptoms [48]. Sleep disturbance and anxiety may have reciprocal causation and form a vicious circle, eventually affecting the maternal physical health and endangering the fetal growth [49]. As a result, healthcare professionals should assess women with twin pregnancies’ sleep conditions regularly, investigate the causes of sleep disturbance, and provide targeted intervention to assist women with twin pregnancies recover to a healthy physical and psychological state. Digital cognitive behavioural therapy (CBT) may be a promising way to treat insomnia symptoms — the main sleep complaint of pregnant women [50].

Notably, in women with twin pregnancies, good previous health status was negatively associated with anxiety and depressive symptoms. Similarly, previous research has found that poor physical health is a major risk factor for perinatal depression [51]. Women expecting twins may experience poorer physical well-being than those expecting a single child [19]. The higher a pregnant woman’s cognitive evaluation of her physical condition, the more confident she will be when confronting the changes, which reflects the close relationship between self-perceived physical health and mental health. Therefore, healthcare providers should pay more attention to the self-reported poor health status of women with twin pregnancies, help them understand the impact of physical health on the pregnancy of twins, correct misperceptions, and promote a sense of self-efficacy in delivery.

Our findings suggest that being under 25 may increase the risk of prenatal depressive symptoms in women with twin pregnancies. A very young age is associated with a low level of resilience[52]. When faced with the enormous physical, psychological and financial challenges brought by twin pregnancy, young women may feel unable to cope with the new situation [53]. Long-standing feelings of helplessness and frustration and a lack of resilience to resist the negative effect of stress may lead to depressive symptoms [51]. Further research into the psychology of this young group and their personal and social resources is warranted. As a low-cost, short-term and effective intervention measure [54], mindfulness-based interventions may be a promising method for these women with twin pregnancies to use daily to improve prenatal depressive symptoms [55].

In this study, one of the associated factors for prenatal depressive symptoms in women with twin pregnancies was the experience of negative life events. The finding is consistent with studies in singleton pregnancies [56, 57]. Negative life events incorporate changes that individuals experience in their social lives that have a negative impact on their mental health in the long term [58].In our study, women with twin pregnancies who reported negative life events during their pregnancy were 3.3 times more likely to have depressive symptoms than those who did not. However, the influence of life events on depression is not straightforward [59]. It has been proposed that social support plays an important role in the relationship between negative life events and depression [60, 61]. Family members, the essential source of social support [62], should provide these women who have conceived twins with sufficient emotional support and living care [63], and should learn to recognize the signs of depression and seek professional help as soon as possible.

Regular physical activity during pregnancy protects women pregnant with twins from depressive symptoms. Physical activity appears to have an inverse relationship with antenatal depressive symptoms [64], and it is a potential non-pharmacological therapy for prenatal depression [65]. However, physical activity recommendations for women with twin pregnancies are limited and impractical [66]. More randomized controlled trials are needed to determine the appropriate physical activity patterns for women with twin pregnancies. By engaging in moderate physical activity such as yoga, women with twin pregnancies may be able to relieve stress and reduce depressive symptoms [67].

This is the first study to report the prevalence of anxiety symptoms and co-morbid anxiety and depressive symptoms in women with twin pregnancies. One strength of this study was investigating associated factors of anxiety and depressive symptoms, including negative life events, sleep disturbance and physical activity in the twin pregnancy population. Moreover, our sample size is comparatively larger than in previous studies. Electronic questionnaires provided a high response rate for this study and enhanced the accuracy of the information. Based on this study, future prospective studies are needed to determine the causal relation between anxiety and depressive symptoms and the associated factors. It would be of great importance to further study the effects of anxiety and depressive symptoms on twin pregnancy outcomes and implement preventative psychological intervention targets on women with twin pregnancies.

There are a few methodological limitations in our study that should be taken into consideration. First, our study sample was obtained through convenience sampling. Our participants, in particular, are primarily city dwellers. Women with twin pregnancies having antenatal examinations in rural hospitals may be more prone to anxiety and depression [68]. So, this study cannot represent the entire population of women pregnant with twins. Secondly, due to the cross-sectional study design, we cannot determine causality in the relationships between our variables. Thirdly, self-reporting questionnaires and the stigma associated with mental health issues [69] may have resulted in an underestimation of the prevalence of antenatal anxiety and depressive symptoms. Fourthly, the prevalence of clinically diagnosed prenatal anxiety and depression in our twin pregnancy population was not determined as mental status examinations were not performed.

## Conclusion

One in three women with twin pregnancies in our sample had symptoms of anxiety or depression, and these symptoms were significantly associated with maternal age, previous health status, sleep disturbance, physical activity, and negative life events. According to the above associated factors, high-quality body-mind interventions such as cognitive behaviour therapy, mindfulness-based intervention, and yoga might contribute to managing anxiety and depressive symptoms and improving the perinatal outcomes of twin pregnancies.

## Data Availability

The datasets used and/or analyzed during the current study are available from the corresponding author on reasonable request.

## Abbreviations

ART: assisted reproduction techniques
BMI: Body Mass Index
RMB: Renminbi
SDS: Self-Rating Depression scale
SAS: Self-Rating Anxiety scale
M: mean
SD: standard deviation
ANOVA: analysis of variance
N: number
OR: Odds Ratio
B: Regression coefficients
df: degree of freedom
P: p-value
CI: Confidence Interval
CBT: Cognitive Behavior Therapy
